# Arthropod steroid hormone (20-Hydroxyecdysone) suppresses IL-1β- induced catabolic gene expression in cartilage

**DOI:** 10.1186/s12906-015-0520-z

**Published:** 2015-01-24

**Authors:** Shiow-Yunn Sheu, Shin-Rong Ho, Jui-Sheng Sun, Ching-Yun Chen, Cherng-Jyh Ke

**Affiliations:** School of Pharmacy, College of Pharmacy, Taipei Medical University, Taipei, Taiwan; Department of Orthopedic Surgery, National Taiwan University Hospital Hsin-Chu Branch, No.25, Lane 442, Sec. 1, Jingguo Rd., Hsin-Chu City, ROC 30059 Taiwan; Department of Orthopedic Surgery, College of Medicine, National Taiwan University, No. 1, Ren-Ai Rd., Taipei, ROC 10051 Taiwan; Institute of Biomedical Engineering, College of Engineering and College of Medicine, College of Medicine, National Taiwan University, Taipei, Taiwan; Biomimetic Systems Research Center, National Chiao Tung University, Hsin-Chu City, Taiwan

**Keywords:** 20-Hydroxyecdysone, IL-1β, Hypoxia, Normoxia, Cartilage destruction, HIF-2α

## Abstract

**Background:**

In osteoarthritis (OA), the imbalance of chondrocytes’ anabolic and catabolic factors can induce cartilage destruction. Interleukin-1 beta (IL-1β) is a potent pro-inflammatory cytokine that is capable of inducing chondrocytes and synovial cells to synthesize MMPs. The hypoxia-inducible factor-2alpha (HIF-2alpha, encoded by Epas1) is the catabolic transcription factor in the osteoarthritic process. The purpose of this study is to validate the effects of ecdysteroids (Ecd) on IL-1β- induced cartilage catabolism and the possible role of Ecd in treatment or prevention of early OA.

**Methods:**

Chondrocytes and articular cartilage was harvested from newborn ICR mice. Ecd effect on chondrocytes viability was tested and the optimal concentration was determined by MTT assay. The effect of HIF-2α (EPAS1) in cartilage catabolism simulated by IL-1β (5 ng/ml) was evaluated by articular cartilage explants culture. The effects of Ecd on IL-1β-induced inflammatory conditions and their related catabolic genes expression were analyzed.

**Results:**

Interleukin-1β (IL-1β) treatment on primary mouse articular cartilage explants enhanced their Epas1, matrix metalloproteinases (MMP-3, MMP-13) and ADAMTS-5 genes expression and down-regulated collagen type II (Col2a1) gene expression. With the pre-treatment of 10^−8^M Ecd, the catabolic effects of IL-1β on articular cartilage were scavenged.

**Conclusion:**

In conclusions, Ecd can reduce the IL-1β-induced inflammatory effect of the cartilage. Ecd may suppress IL-1β- induced cartilage catabolism via HIF-2α pathway.

## Background

Osteoarthritis (OA) is a primarily degenerative disease of the joints; it is an enormous and expensive public health problem. The incidence of OA increases with age, but a variety of potential factors (such as: hereditary, developmental, metabolic, and mechanical factors) may initiate processes leading to loss of cartilage [1]. The high prevalence, together with the high degree of disability, makes this disease a substantial burden for societies [2]. There are no effective medical therapies to prevent cartilage destruction and the associated bony changes in the osteoarthritic joint.

Chondrocytes hypertrophy does occur in osteoarthritic cartilage. The articular chondrocytes hypertrophy partially reflexed that the degrading and remodeling of the extracellular matrix promote pathologic cartilage calcification. An imbalance between anabolic and catabolic factors within chondrocytes may cause osteoarthritic cartilage destruction [3-5]. The hypoxia-inducible factor-2alpha (HIF-2α, encoded by Epas1) is a catabolic transcription factor in the osteoarthritic process. HIF-2α directly induces chondrocytes expression of genes encoding catabolic factors, such as matrix metalloproteinases (MMP1, MMP3, MMP9, MMP12 and MMP13), aggrecanase-1 (ADAMTS4), nitric oxide synthase-2 (NOS-2) and prostaglandin-endoperoxide synthase-2 [3,4]. According to this paradigm, a stress-induced increase in the activity of HIF-2α will overshadow the beneficial effects of the closely related HIF-1α, then push the chondrocytes in the joint toward a more differentiated state known as hypertrophy, which then drives osteoarthritis changes [6,7].

Ecdysteroids (Ecs) are steroids found in invertebrates and plants [8,9]. The possible active ingredient best known and investigated is 20-hydroxy-Ecdysone (β-Ecdysone=Ecd) [10]. In mammals, Ecd has protein anabolic effects and increases muscle mass of unexercised and exercised muscle [11,12]. In ovariectomized rats, pure Ecd supplementation significantly increased in the thickness of joint cartilage and presented beneficial effects in bones. This result provides evidence that pure Ecd may be of value in the prevention and treatment of osteoporosis and osteoarthritis [13-15].

The Ecd is frequently used by body builders and from these users it is known that Ecd has no known adverse effects even when taken up in gram (~1 g) quantities [16]. The selective, rationally designed biologic drugs that slow joint destruction must be in clinical use earlier before that can happen. Such modality should be used to the onset of chondrocytes hypertrophy and catabolism and before loss of cartilage progresses to the point where it is clinically beyond a ‘point of no return’. The purpose of this study is to validate the effects of Ecd on IL-1β- induced cartilage destruction and also the possible role of Ecd in treatment or prevention of early OA.

## Methods

### Chemicals and reagents

20-Hydroxyecdysone (Edc) was obtained from Enzo Life Sciences; New York, USA. Edc stock solutions (10^−2^M) were prepared in phosphate buffer solution (PBS, Sigma Chemical, St. Louis, MO, USA) and stored at −20°C.

### Isolation of chondrocytes

Chondrocytes were obtained from newborn ICR mice (3 days-old; the laboratory center of the Medical College, National Taiwan University). Briefly, articular cartilages was dissected under sterile conditions, washed and then digested with 0.2% collagenase [17]. After 7 days in monolayer culture, total cell yield of chondrocytes was counted and their cell viability was tested with trypan-blue exclusion assay. The surgical procedures and experimental protocols were approved and under supervision by the Medical College’s Animal Research Committee of the National Taiwan University and have been carried out in accordance with the Declaration of Helsinki.

### 3-[4,5-dimethylthiazol]-2,5-diphenylterazolium bromide assay (MTT assay)

Chondrocytes (1×10^4^ cells/well) were seeded into 96-well plates. After 2 days of incubation, α-MEM (with 1% FBS) containing 10^−6^M to 10^−10^M Ecd was added 1 day before test. The 3-(4, 5-dimethylthiazolyl-2)-2, 5-diphenyltetrazolium bromide (MTT; Sigma Co., St. Louis, MO, USA) assay for cell viability was performed on the 1^st^, 3^rd^, 7^th^, 10^th^ day of culture. During the experiment, the treatment (including medium and medication) was changed every 3 days and fresh Ecd was added at each media change. The level of mitochondrial activity of the bone cells after Ecd treatments were determined by colorimetric assay, which detects the conversion of MTT to insoluble formazan. The plates were read on the ELISA reader (Spectra max 340, molecular Devices; CA, USA) at a wavelength of 570 nm.

### The role of HIF-2α (EPAS1) in cartilage destruction

Chondrocytes were seed into eight 12-well plates at a density of 4×10^4^ cells/well. After 1 days of incubation, the cells were washed twice with PBS solution and then IL-1β (5 ng/ml) was added to α-MEM (with 1% FBS) for test. The Epas1 and Col2a1 genes expression after IL-1β treatment were analyzed.

### Articular cartilage explants culture

Newborn ICR mice (3 days-old) were obtained from the laboratory center of the Medical College, National Taiwan University. Under sterile condition, fibrous tissues were removed, the articular cartilage explants were washed and then seeded to α-MEM (containing 1% FBS) one day before test. After 1 day’s pre-incubation with/ or without Ecd (20-Hydroxyecdysone: 10^−8^M), IL-1β (5 ng/ml) was added for further test.

### The effects of Ecd on IL-1β-induced inflammatory conditions

The articular cartilage explants were seeded to α-MEM containing 1% FBS on the 1 day before test, samples were incubated with IL-1β (5 ng/ml) for 48 hours. To evaluate the effects of Ecd under IL-1β-induced inflammatory conditions, the Ecd (20-Hydroxyecdysone: 10^−8^M) was added 24 hours before or after the adding of IL-1β treatment; the articular cartilage explants were incubated at hypoxic (3.5% O_2_) or normoxic conditions.

### Gene expression analysis

After treatment, RNA for analysis was isolated at the time point on 0, 3, 6, 12, 24, 36 hours after Ecd addition. Briefly, the cell cultures were washed with PBS, total RNA was extracted and then cDNA reversely transcripted. PCR amplification was performed by the Light Cycler FastStart DNA Master SYBR Green I (Roche, Mannheim, Germany). The amplification was performed in a Roche Light Cycler 2.0 instrument under the following condition: initial denaturation at 95°C for 10 min and 45 cycles of denaturation at 95°C for 5 sec, annealing at 55°C for 5 sec, and extension at 72°C for 8 sec. For each genes analysis, the experiments were repeated at least three times. The internal standard gene used was β-actin and the genes been analyzed were listed in Table 1.

**Table 1:** **Primers sequences for reverse transcription–polymerase chain reaction (RT–PCR)**

**Gene name**	**Primer(upper:sense primer)**
**(Gene bank)**	**(Lower: antisense primer)**
Col2a1	F: 5'-TGAAGACATCCGCAGCCCC -3'
(NM_031163)	R: 5'-ATAATGGGAAGGCGGGAGG-3'
MMP-3	F: 5'-CCTTTTGATGGGCCTGGAA-3'
(XM_284824)	R: 5'-ATCGTCAAAGTGAGCATC-3'
MMP-13	F: 5'-CTTGTGTTTGCAGAGCACTA -3'
(NM_008607.1)	R: 5'-ACTGTGGAGGTCACTGTAGA-3'
ADAMTS5	F: 5'-GCCATTGTAATAACCCTGCACC-3'
(XM_122978.1)	R: 5'-TCAGTCCCATCCGTAACCTTTG-3'
EPAS1	F: 5'-CGAGAAGAACGACGTGGTGTTC-3'
(NM_010137.2)	R: 5'-GTGAAGGCGGGCAGGCTCC-3'
β-Actin	F: 5’-CAGTTCGCCATGGATGACGAT3’
(NM_007393)	R: 5’-CATAGCTCTTCTCCAGGGAG-3’

### Statistical analysis

All experiments were performed at least in triplicate. Results were expressed as mean ± standard deviation of these experiments and statistically analyzed by Two-way ANOVA. Statistical significance by Dunnett’s test was set at p < 0.05 between the means of the control and test groups.

## Results

### Ecd treatment enhance chondrocytes viability

**Figure 1:**
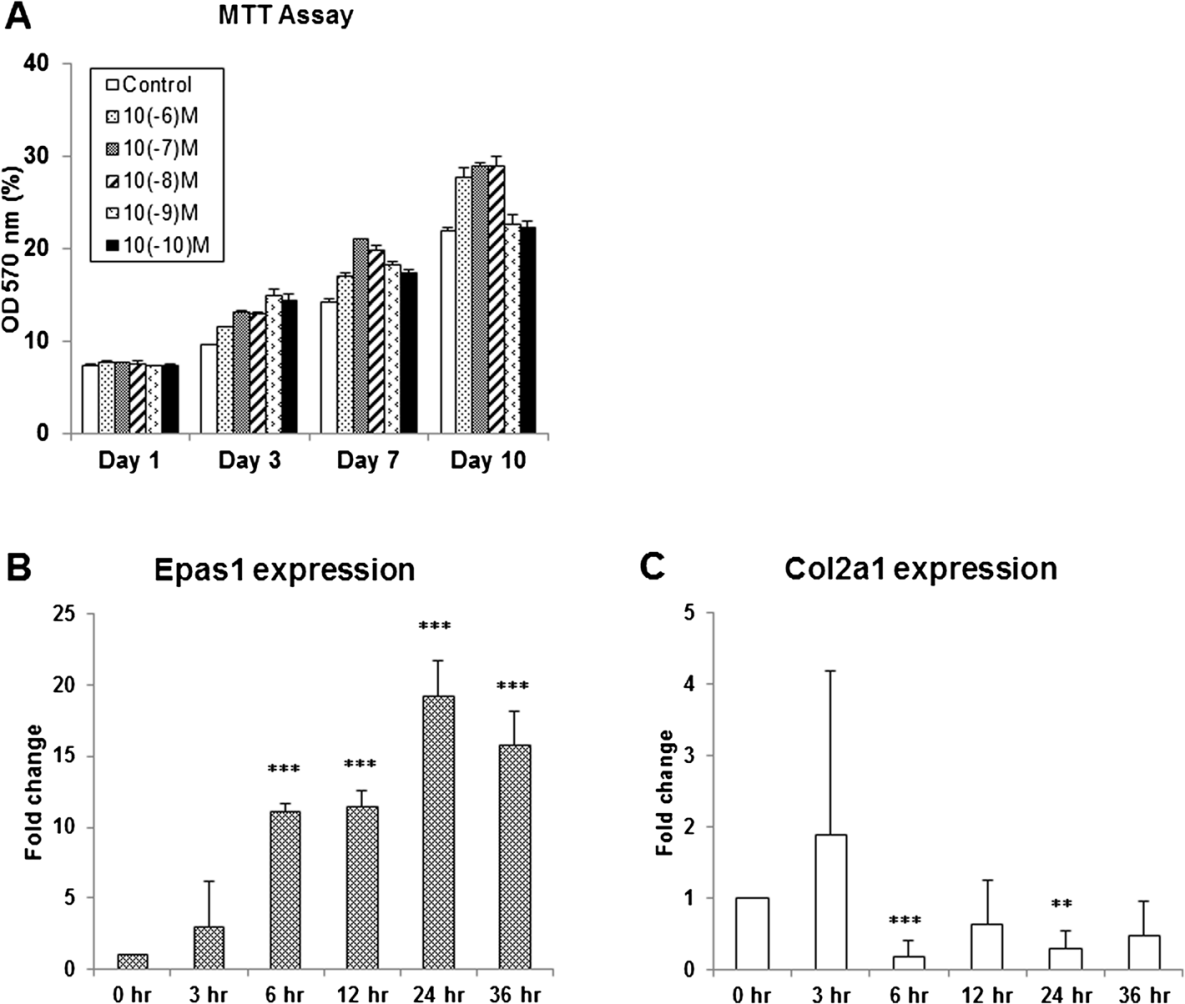
**Effect of Ecd and IL-1 beta on chondrocytes. A** Ecd treatment enhance chondrocytes viability. Chondrocytes at every concentration proliferate actively, with significant statistical differences observed between chondrocytes treated with Ecd and that of the control. Chondrocytes’ viability reaches its maximal beneficial effect at 10^−8^M Ecd after 10 days of culture. **B** and **C** IL-1β induce HIF-2α (encoded by Epas1) gene expression in chondrocytes. In this study, mouse articular chondrocytes are treated with IL-1β cytokines. The results showed that IL-1β can enhance Epas1 gene expression, while suppress collagen type II (Col2a1) gene expression. (Each value is the mean ± standard deviations; n = 8; *:p<0.05; **:p< 0.01; ***:p < 0.001).

The effect of Ecd on chondrocytes viability was examined at Ecd concentrations of 0, 10^−6^, 10^−7^, 10^−8^, 10^−9^, and 10^−10^M at 1, 3, 7, and 10 days of culture (n = 8; Figure 1A). In this study, there is significant difference observed between treated cells with that of the control (p < 0.05). We chose 10^−8^ M Ecd for the further evaluation because there is maximal viability of chondrocytes at this concentration (p < 0.01) at the 10th day’s culture.

### IL-1β induce HIF-2α (encoded by Epas1) gene expression in chondrocytes

IL-1β can induce Epas1 gene expression in chondrocytes [4]. In this study, interleukin-1β (IL-1β) treatment of primary mouse articular chondrocyte cultures enhance Epas1 mRNA expression, while suppress collagen type II (Col2a1) gene expression (Figure 1B, C).

### Ecd pre-treatment reduce the catabolic effect of IL-1β on the cartilage explants

**Figure 2:**
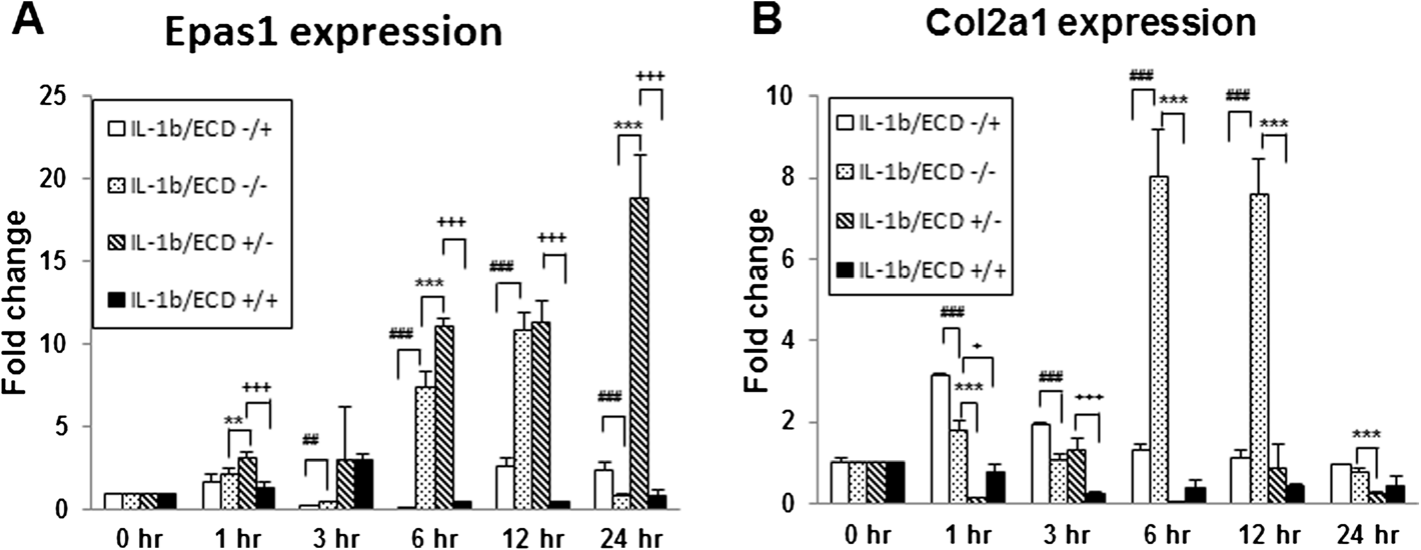
**Regulatory mechanisms of Epas1 expression in articular cartilage explants under IL-1β treatment.** In this study, mouse articular cartilage explants are treated with IL-1β cytokines. The results showed that IL-1β can enhance Epas1 gene expression **(A)**, and suppress collagen type II (Col2a1) gene expression **(B)**. The pretreatment of 10−8M Ecd can effectively eliminate the Epas1 gene expression enhanced by IL-1β treatment. (Each value is the mean ± standard deviations; n = 8; *:p<0.05; **:p< 0.01; ***:p < 0.001).

**Figure 3:**
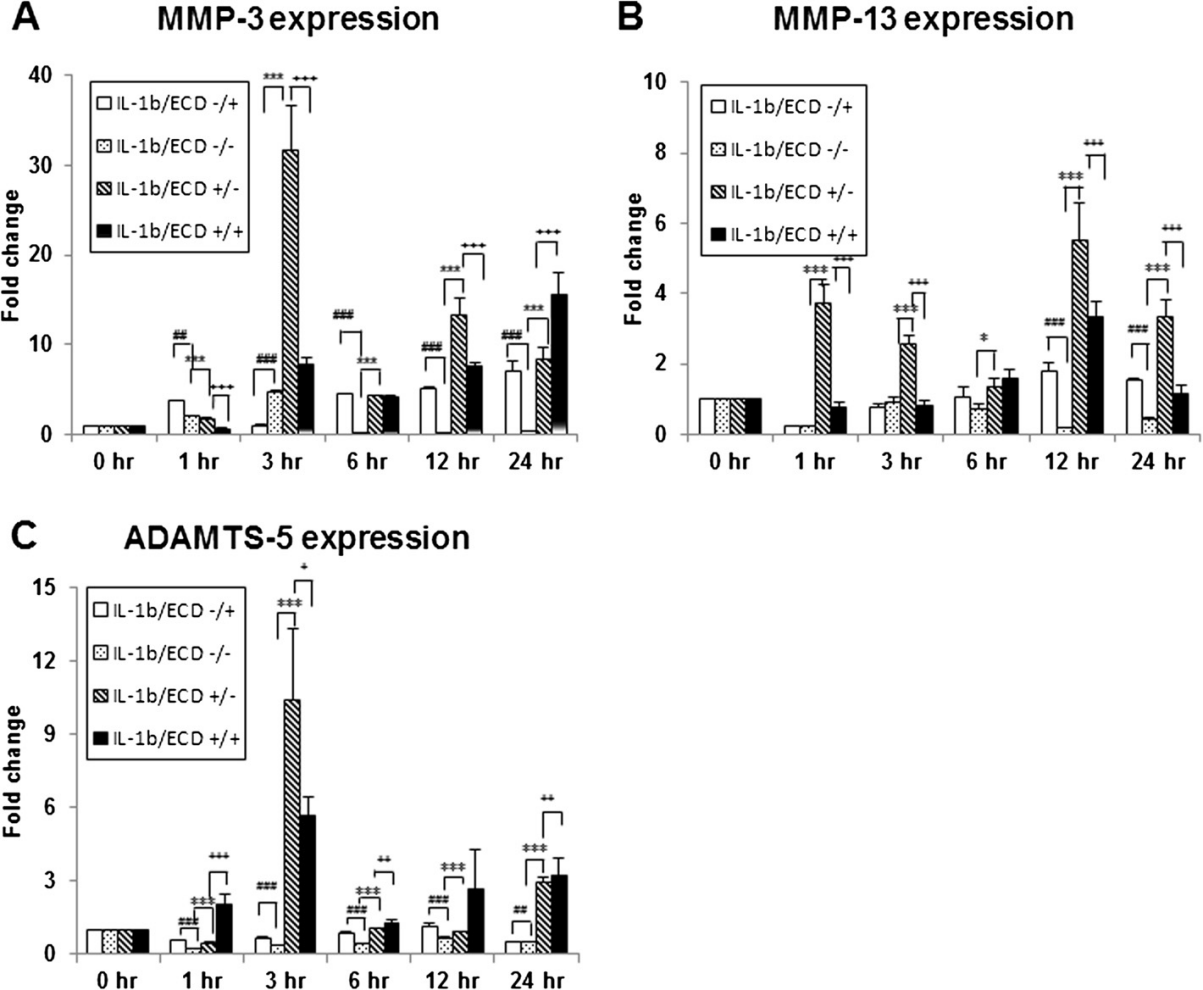
**Ecd pre-treatment reduce the catabolic effect of IL-1β on the cartilage explants.** Treatment of primary cultures of mouse articular cartilage explants with recombinant mouse IL-1β protein specifically induce the expression of MMP3 **(A)**, MMP13 **(B)** and ADAMTS-5 **(C)** genes expression; with the pretreatment of 10−8M Ecd, the catabolic factor genes upregulated by IL-1β treatment are eliminated. (Each value is the mean ± standard deviations; n = 8; *:p<0.05; **:p< 0.01; ***:p < 0.001).

The catabolic effect of IL-1β on cartilage explants are mediated via HIF-2α–induced expression of MMP3, MMP13 and ADAMTS-5. The IL-1β related gene expression of primary mouse articular cartilage explants are examined. Same as noted above, IL-1β enhances Epas1 mRNA expression and suppresses collagen type II (Col2a1) gene expression. With the pretreatment of 10^−8^M Ecd, it can scavenge IL-1β effect on Epas1 gene expression, but there is also no effect on Col2a1 gene expression (Figure 2). IL-1β treatment also specifically up-regulate MMP3, MMP13 and ADAMTS-5 genes expression (Figure 3). The pre-treatment of 10^−8^M Ecd can effectively down-regulate the catabolic genes expression. Ecd can reduce the catabolic effect of IL-1β on the cartilage explants; Ecd has protective effects on articular cartilage by inhibiting Epas1, MMP-3, MMP-13 and ADAMTS-5 genes expression.

### Ecd protect cartilage explants both in hypoxic and normoxic condition

Under hypoxic condition, the expression of Epas1 was higher; Ecd could suppress the IL-1β induced Epas1 and ADAMTS-5 genes expression both in hypoxic and normoxic conditions.

**Figure 4:**
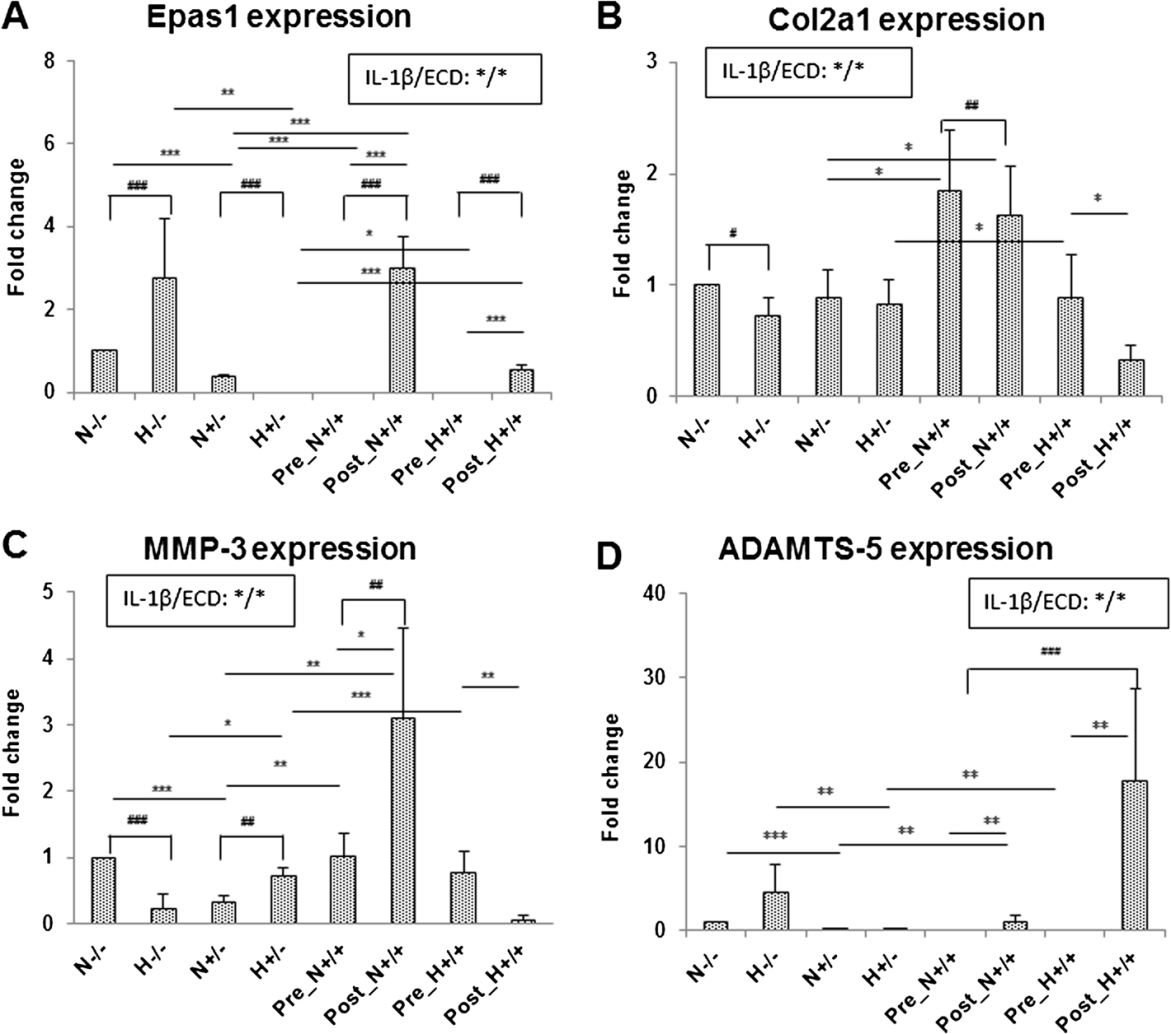
**Ecd protect cartilage explants both in hypoxic and normoxic conditions.** The Epas 1 gene expression is enhanced under hypoxic condition. With the pre-treatment of 10−8M Ecd, it can effectively eliminate the IL-1β enhanced Epas1 gene expression **(A)**. The Col2a1 gene expression was stationary under normoxic condition **(B)**. Under hypoxic condition, the pretreatment of 10−8M Ecd can effectively eliminate the IL-1β down-regulated Col2a1 gene expression. Under hypoxic condition, IL-1β mediates HIF-2α–induced ADAMTS-5 gene expression of articular cartilage. The pretreatment of 10−8M Ecd can effectively down-regulate MMP3 gene expression only under normoxic condition **(C)**; while down-regulate ADAMTS5 genes expression both in normoxic and hypoxic conditions **(D)**. (Each value is the mean ± standard deviations; n = 8; *:p<0.05; **:p< 0.01; ***:p < 0.001).

Under hypoxic condition, the Epas 1 gene expression is enhanced. Treatment of 10^−8^M Ecd eliminates the IL-1β-enhanced Epas1 gene up-regulation; this effect exists only when Ecd was added before the treatment of IL-1β (Figure 4A). The Col2a1 gene expression is stationary under normoxic condition; while in hypoxic condition, Ecd pre-treatment eliminates the IL-1β-induced Col2a1 gene down-regulation (Figure 4B). Ecd does have protective effect on Col2a1 gene expression under hypoxic condition.

Under hypoxic condition, IL-1β mediates HIF-2α–induced up-regulation of ADAMTS-5 gene (but not MMP3 or MMP13 genes) in articular cartilage (Figure 4C and D); there was no significant expression on MMP13 gene under hypoxic condition (data not shown). The pre-treatment of 10^−8^M Ecd can effectively down-regulate MMP3 gene expression only under normoxic condition; while down-regulate ADAMTS5 genes expression both in normoxic and hypoxic conditions (Figure 4D). MMP3 and MMP13 are probably not key catabolic factors under hypoxic condition.

## Discussion

Osteoarthritis is a chronic degenerative disorder that mostly affects the articular cartilage. Joint pain, stiffness, impaired mobility and reduced quality of life is the commonest presentation of this disease in elderly people [18]. Osteoarthritic cartilage destruction is initiated by the imbalance of anabolic and catabolic factors in cartilage [19]. These factors include pro-inflammatory cytokines that have key roles in the catabolic reactions of arthritic cartilage [20]. Pro-inflammatory cytokines are capable of inducing HIF-2α; HIF-2α then causes cartilage destruction by regulating crucial catabolic genes, such as matrix metalloproteinases (MMP1, MMP3, MMP9, MMP12 and MMP13), aggrecanase-1 (ADAMTS4), nitric oxide synthase-2 (NOS2) and prostaglandin-endoperoxide synthase-2 (PTGS-2) through the expression and activity of Epas1 in chondrocytes [4]. Pure ecdysteroids (Ecs) supplementation significantly increased in the thickness of joint cartilage and also presented an overview of beneficial effects in bones and may be of value in the prevention and treatment of osteoporosis and osteoarthritis [13-15]. In this study, we validate that Ecd inhibit IL-1β- induced cartilage catabolism via HIF-2α pathway.

Cartilage tissue is characterized by its high content of aggrecans, which exists in association with hyaluronic acid and links proteins to form proteoglycan aggregates. In osteoarthritis (OA), the loss of proteoglycans, the mineralization of the extracellular matrix (ECM) and the hypertrophic differentiation of the chondrocytes constitute hallmarks of this disease [21]. The balance between ECM synthesis and degradation is often associated with a dedifferentiation of chondrocyte and/or an enhanced apoptosis [22]. During OA, aggrecans are among the first matrix constituents to be affected, as they are progressively depleted from cartilage in parallel with the severity of the disease [23]. Chondrocytes try to compensate for the loss of proteoglycans by increasing their production. At a certain stage in the development of OA, however, chondrocytes are unable to fully compensate for proteoglycan loss, resulting in a net loss of matrix. The increased loss of aggrecans fragments is mostly due to the action of both MMPs and aggrecanases [24].

IL-1β is a potent pro-inflammatory cytokine that is capable of inducing chondrocytes and synovial cells to synthesize MMPs [14]. In this study, we demonstrated that inflammatory cytokine (interleukin-1β: IL-1β) does enhance the Epas1 gene expression and down-regulated collagen type II (Col2a1) gene expression in chondrocytes culture (Figure 1); while the pre-treatment of Ecd can scavenge the catabolic effects of IL-1β on chondrocytes (Figure 2).

In the early phases of OA, MMP-13 is predominantly expressed in the lower intermediate and deep layers of human and mouse cartilage, whereas aggrecanases are expressed in the superficial cartilage [25,26]. The degradation of aggrecan by aggrecanases has gained special interest, because it constitutes an early event in osteoarthritic cartilage damage and is followed by the irreversible loss of collagens [27]. Aggrecanases belong to the ADAMTS (a disintegrin and metalloproteinase with thrombospondin motifs) family of metalloproteinases. During OA, ADAMTS-4 (aggrecanase 1) [28] and ADAMTS-5 (aggrecanase 2) [29,30] have been implicated prominently in cartilage breakdown. In spite of a large variability of expression levels among different patients, MMP-3 appeared to be strongly expressed in normal and early degenerative cartilage and down-regulated in the late disease stages. In the late stages of cartilage degeneration, MMP-2 and MMP-13, which were up-regulated [31]. On primary mouse articular cartilage explants cultures, interleukin-1β (IL-1β) treatment enhanced their Epas1, MMP-3, MMP-13 and ADAMTS-5 genes expression and down-regulated collagen type II (Col2a1) gene expression. With the pre-treatment of 10^−8^M Ecd, the catabolic effects of IL-1β on articular cartilage were scavenged (Figure 3).

Hypoxic signaling plays an essential role in maintaining oxygen homeostasis and cell survival. Recent studies have identified an important role for HIF-1 and HIF-2 in the regulation of skeletal development, bone formation, and regeneration, as well as joint formation and homeostasis. In addition, over-expression of HIF-1 and HIF-2 is clinically associated with osteosarcoma and osteoarthritis. Together, these findings implicate hypoxic signaling as a central regulator of skeletal biology and disease [32]. It is reported that HIF-2α (like IL-1β) does not induce transcription of ADAMTS5 [3,4]. Instead, IL-1β and certain other inflammatory mediators promote activation of latent ADAMTS-5, as does hypertrophic differentiation in chondrocytes [3,4]. In this study, we demonstrated that IL-1β (Epas1 or HIF-2α) induce articular cartilage inflammation and manifested as up-regulation of MMP-3, MMP-13 and ADAMTS-5 under normoxic status; Ecd has protective effects on articular cartilage by inhibiting Epas1, MMP-3, MMP-13 and ADAMTS-5, but there is no ability to recover the cartilage phenotype as Col2a1 gene was not affected (Figure 4). Our study also demonstrated that ADAMTS-5 is the main mediators of the inflammatory cytokine involved under hypoxic status, (Figure 4).

**Figure 5:**
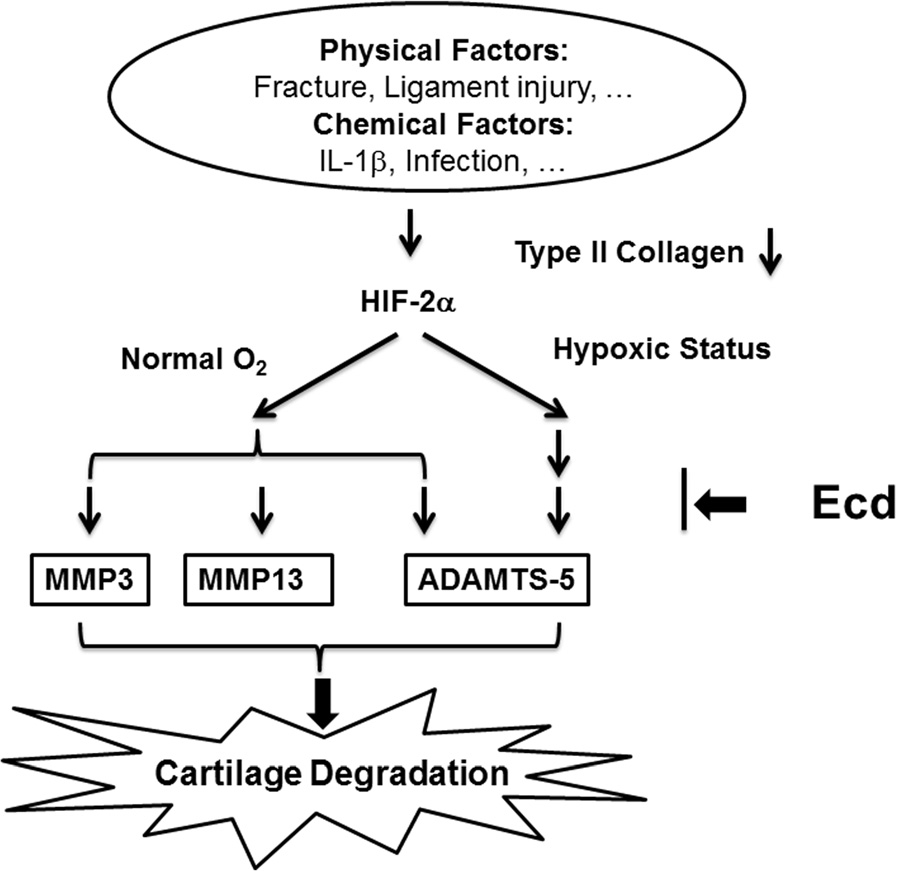
**Molecular mechanism of Ecd chondroprotective effect.** Both physical and chemical factors may induce cartilage degradation via IL-1β/ HIF-2α pathway; 20-Hydroxyecdysone (Ecd) does have chondroprotective effect and can inhibit IL-1β- induced cartilage catabolism via HIF-2α pathway.

There is no cure for osteoarthritis. By piecing together the molecular events that drive the progression of this debilitating disease, recent studies put hypoxia-inducible factor-2α (HIF-2α) in the driver’s seat, opening up new avenues for early detection and treatment [6]. The findings of our study are consistent with the observations that inflammation of articular cartilage stimulates ADAMTS5 activation. The ADAMTS5 activation directly and also indirectly by promoting expression of MMP-3, MMP13 which not only acts on ADAMTS5 but also degrades certain cartilage extracellular matrix constituents under normoxic status. Under the hypoxic status, the inflammation of articular cartilage mainly stimulates ADAMTS5 activation. Hence, synergy between different pathways, both stimulated by HIF-2α is a core feature of osteoarthritis chondrocyte biology (Figure 5). This aspect of the model is important, because some therapies in development have focused on ADAMTS5, but other pathways may offer new means to target ADAMTS5 [33]. In summary, IL-1β enhanced Epas1 mRNA levels, with suppression of collagen type II (Col2a1) expression.

## Conclusion

20-Hydroxyecdysone (Ecd) does have chondroprotective effect and Ecd has protective effects on articular cartilage by inhibiting epas1, MMP-3, MMP-13 and ADAMTS-5. Ecd can inhibit IL-1β- induced cartilage destruction via HIF-2α pathway. However, further researches by *in-vivo* HIF-1alpha gene knock-down and translational medicine are mandatory for the possible clinical application in the future.
